# Parental Reports of Infant and Child Eating Behaviors are not Affected by Their Beliefs About Their Twins’ Zygosity

**DOI:** 10.1007/s10519-016-9798-y

**Published:** 2016-07-12

**Authors:** Moritz Herle, Alison Fildes, Cornelia van Jaarsveld, Fruhling Rijsdijk, Clare H. Llewellyn

**Affiliations:** 1 Cancer Research UK Health Behaviour Research Centre, Department of Epidemiology & Public Health, University College London, London, WC1E 6BT UK; 2 Department for Health Evidence & Department of Primary and Community Care, Radboud University Medical Center, Nijmegen, The Netherlands; 3 Social, Genetic, and Developmental Psychiatry Centre, Institute of Psychiatry, King’s College London, London, UK

**Keywords:** Parental bias, Twin research, Child development, Misclassified zygosity, Eating behaviors, Heritability

## Abstract

Parental perception of zygosity might bias heritability estimates derived from parent rated twin data. This is the first study to examine if similarities in parental reports of their young twins’ behavior were biased by beliefs about their zygosity. Data were from Gemini, a British birth cohort of 2402 twins born in 2007. Zygosity was assessed twice, using both DNA and a validated parent report questionnaire at 8 (SD = 2.1) and 29 months (SD = 3.3). 220/731 (8 months) and 119/453 (29 months) monozygotic (MZ) pairs were misclassified as dizygotic (DZ) by parents; whereas only 6/797 (8 months) and 2/445 (29 months) DZ pairs were misclassified as MZ. Intraclass correlations for parent reported eating behaviors (four measured at 8 months; five at 16 months) were of the same magnitude for correctly classified and misclassified MZ pairs, suggesting that parental zygosity perception does not influence reporting on eating behaviors of their young twins.

## Introduction

Over the past century the Twin Method has been used to investigate genetic and environmental contributions to variation in complex human traits. Researchers have been using this methodology to examine a wide spectrum of aspects of human life accumulating in a total of 17,804 investigated traits, spanning disease, to behavior to opinion. Twin research is conducted worldwide and 14,558,903 twins are currently included in a multitude of studies (Polderman, et al. 2015).

The classic Twin Method is based on comparing the correlations or concordance rates of traits between monozygotic (MZ) and dizygotic (DZ) twin pairs. MZs are genetic clones of one another, sharing 100 % of their genes, whereas DZs share on average only 50 % of their segregating genes. Importantly, both types of twins share their environments to a similar extent. For example, both types of twins are gestated together in the same uterus, and are raised together in one family. Any difference in resemblance between MZ and DZ pairs is therefore assumed to reflect genetic differences only. The univariate method can also be extended to understand if multiple traits share a common etiology, and to establish genetic and environmental contributions to trait stability and change over time (Rijsdijk and Sham 2002; van Dongen et al. 2012).

One of the criticisms of parent reported measures of young twin behavior is that parents are biased by their belief about their twins’ zygosity. For example, it is possible that parents score their twins more similarly if they believe them to be identical, or more differently if they believe them to be non-identical. If this is true, heritability estimates for these traits will be inflated because heritability is estimated by doubling the difference between the MZ and DZ correlations. This bias can be tested for directly by taking advantage of the fact that many parents are mistaken about their twins’ zygosity—the so-called ‘misclassified zygosity design’. Many parents of MZs mistakenly believe them to be DZs (van Dongen et al. 2012). This often results from parents being misinformed by health professionals based on prenatal scan observations or at the twins’ birth if the MZ twins are dichorionic (Ooki et al. 2004). Researchers can take advantage of parental misclassification of zygosity to examine if twin correlations differ for MZs who are correctly and incorrectly classified by parents (the same approach can be used to test for differences between correctly and incorrectly classified DZs, although this occurs much more rarely (van Jaarsveld et al. 2012)). If the correlations for correctly and incorrectly classified MZ pairs are of the same magnitude, it is unlikely that parents are biased in their reporting by their belief about their twins’ zygosity.

Most previous studies using the ‘misclassified zygosity design’ have relied on self-reported zygosity by the twins themselves in order to investigate if their perception of their zygosity shapes their environmental exposure—testing the so-called ‘equal environments assumption’. Results from these studies have suggested that identical twins correlate highly on behavioral traits regardless of their believed zygosity status (Scarr and Carter-Saltzman 1979; Goodman and Stevenson 1989; Xian et al. 2000; Gunderson et al. 2006). This study uses a novel application of the ‘misclassified zygosity design’ to test for parental bias in reporting of a range of eating behaviors in infancy and early childhood.

## Materials and methods

### Sample

Data came from Gemini, a population-based British birth cohort of 2402 families with twins born in 2007 in England or Wales (van Jaarsveld et al. 2010). Ethical approval was granted by the University College London Committee for the Ethics of non–National Health Service Human Research. Participants included 816 families with opposite-sex twin pairs (DZ by default), and 1586 with same-sex twin pairs. Parents of same-sex twin pairs completed a 20-item Zygosity Questionnaire at baseline (Q1), when the twins were on average 8 months old (SD = 2.1, range 4.1–16.7 months) (Price et al. 2000). In addition, 934 families (58.9 %) completed the same questionnaire on a second occasion (Q2) when the twins were on average 29 months old (SD = 3.3, range 22.9–47.6 months). A total of 1127 families had provided DNA samples for both twins, of which 81 pairs were randomly selected for zygosity testing. Parents also completed measures of infant and child eating behavior when the twins were on average 8 months (SD = 2.1, range 4.1–16.7 months) and 16 months (SD = 1.2, range 13.4–27.4 months) old respectively. Only data from same-sex twin pairs were used in the analyses in this study. Twin pairs with missing or inconclusive zygosity data were excluded.

### Zygosity questionnaire

The items in the zygosity questionnaire relate to physical resemblance including: general similarity; similarity of specific features such as hair color and texture, eye color, ear lobe shape; timing of teeth coming through; and ease with which parents, friends and other family members can distinguish the twins. Other items ask about blood type, health professional’s opinion, and the parents’ own opinion on zygosity (Price et al. 2000). The zygosity questionnaire is scored by adding up the scores obtained for each question and dividing the total by the maximum possible score based upon the number of questions answered to create a value between 0 and 1. Lower scores indicate greater intra-pair similarity with zero representing maximal similarity and one maximal dissimilarity. Scores <0.64 were classified as MZ, scores >0.70 were classified as DZ, and scores between 0.64 and 0.70 were coded as ‘unclear’ zygosity, as described by Price et al. (2000).

### DNA genotyping

Hyper-variable minisatellite DNA probes are used to detect multiple tandem-repeat copies of 10–15 base pair sequences scattered throughout the human genome (Hill and Jeffreys 1985; Jeffreys et al. 1985). In MZ twins, the bands are identical, but they differ in DZ twins. 1127 families provided DNA using saliva samples for both twins. To validate the zygosity questionnaire, DNA was analyzed in a randomly selected sample of 81 twin pairs. In addition, some families elected to have their DNA used for zygosity testing (n = 118) and we tested a further 111 pairs who could not be classified using questionnaire data (or did not complete the second questionnaire) and who had provided DNA samples. Of these, 41 pairs recorded a mismatch between the two questionnaires; 59 pairs were classified as uncertain at one or both time points; and 24 pairs were missing the second zygosity questionnaire. A total of 310 pairs were therefore zygosity-tested using DNA. We also assessed the concordance between the 8- and 29-month zygosity questionnaire classification, with the DNA-classified zygosity for all of these pairs for whom DNA was available, to evaluate the relative accuracy of the 8 versus 29-month questionnaire. However, this sample largely included pairs who were not easily classified using the questionnaire.

### Parental beliefs about zygosity

When the twins were approximately 8 months old (mean = 8.17, range 4.01–20.3) parents were asked to classify their twins as MZ or DZ, using the question: “Do you think your twins are identical? (‘yes’ or ‘no’)”. Parental classifications were available for 1565 same-sex twin pairs. The same question was asked again when the twins were 29 months old (SD = 3.3, range 22.9–47.6 months) old, and 898 parents responded. To gain further insight into how beliefs about zygosity are formed, parents were also asked if they had ever received zygosity information regarding their twins from health professionals, using the question: “Have you been told by a health professional that your twins are identical or non-identical?”.

### Baby eating behavior questionnaire

The Baby Eating Behavior Questionnaire (BEBQ) (Llewellyn et al. 2011) was completed by parents when the twins were 8 months old (mean = 8.17, SD = 2.18) old. The BEBQ measures four distinct eating behaviors during the period of exclusive milk-feeding (the first 3 months after birth, before any solid food has been introduced) that have been associated with infant weight gain (van Jaarsveld et al. 2011, 2014). Satiety Responsiveness (SR) measures an infant’s ‘fullness’ sensitivity (e.g. how easily he or she gets full during a typical milk feed). Food Responsiveness (FR) assesses how demanding an infant is with regard to being fed, and his or her level of responsiveness to cues of milk and feeding (e.g. wanting to feed if he or she sees or smells milk). Enjoyment of Food (EF) captures an infant’s perceived liking of milk and feeding in general (e.g. the extent of pleasure experienced while feeding). Slowness in Eating measures the speed with which an infant finishes a typical milk feed (e.g. his or her overall feeding pace).

Parents used a 5-point Likert scale (1 = Never, 5 = Always) to report how frequently they observed their infant demonstrate a range of eating behaviors characteristic of each scale. Numbers of items per scale and example items are shown in Table 1. The BEBQ is an adaptation of the Child Eating Behavior Questionnaire (CEBQ), and has been validated in a different sample (Mallan et al. 2014). Mean scores for each subscale were only calculated if a minimum of items were entered (2/3, 3/4 or 4/5).

**Table 1:** Scales, number of items per scale and example items for the BEBQ and the CEBQ

	Number of items	Example item
*BEBQ*		
FR	5	My baby was always demanding a feed
SR	3	My baby found it difficult to manage a complete feed
SE	4	My baby took more than 30 min to finish feeding
EF	4	My baby loved milk
*CEBQ-T*		
FR	4	My child is always asking for food
SR	5	My child gets full up easily
SE	4	My child takes more than 30 min to finish a meal
EF	4	My child loves food
EOE	3	My child eats more when annoyed
FF	6	My child refuses new foods at first

### Child eating behavior questionnaire (Toddler)

The Child Eating Behavior Questionnaire for toddlers (CEBQ-T) was completed by parents when their children were 16 months old (Mean = 15.8, SD = 1.2). In keeping with the BEBQ parents used the same 5-point Likert scale (1 = Never, 5 = Always) to rate the twins for six distinct eating behaviors. The CEBQ-T measures the same four traits as the BEBQ (SR, FR, EF and SE), in relation to food rather than milk, as well as two other eating behaviors that have been associated with child weight. Food Fussiness (FF) measures a child’s tendency to be highly selective what foods he or she is willing to eat, as well as the tendency to refuse to try new food items. Emotional Overeating (EOE) captures a child’s the tendency to eat more in response to stress and negative emotions. The number of items per scale and example items are shown in Table 1.

The CEBQ-T is a modified version of the validated CEBQ (Wardle et al. 2001) which has been validated against laboratory-based measures of eating behaviors (Carnell and Wardle 2007). The CEBQ has been widely used to establish relationships between eating behavior and pediatric weight status (Carnell and Wardle 2007; Viana et al. 2008; Webber et al. 2009; Mallan et al. 2013; Domoff et al. 2015). The CEBQ-T was modified to be appropriate for toddlers. The majority of the items between the CEBQ and the CEBQ-T are identical. However, the emotional undereating and desire to drink scale from the original CEBQ were removed as mothers reported their children not to engage in these behaviors. Furthermore, the wording of some EOE items was modified. Words describing the child’s mood were changed to make them more age appropriate (‘worried’, ‘annoyed’ and ‘anxious’ were replaced for ‘irritable’, ‘grumpy’ and ‘upset’). One item of the SR scale was extended from ‘my child always leaves food on his/her plate at the end of a meal’ to ‘my child always leaves food on his/her plate or in the jar at the end of a meal’. Finally the item ‘If given the chance, my child would always have food in his/her mouth’ was omitted from the FR scale. Similar to the BEBQ, means for the CEBQ-T subscales were calculated if majority of the items were answered (2/3, 3/4, 4/5 or 4/6).

### Analyses

#### Researcher classification of zygosity

Zygosity results from the two questionnaires were compared in 934 pairs who had data for both, to assess the test–retest correlation and percentage agreement. The questionnaire results were compared to DNA results in the random sub-sample of 81 pairs. Analyses were performed using SPSS 22 for Windows.

#### Comparison of twin correlations for correctly and incorrectly classified pairs

Concordance and discordance between parents’ beliefs about their twins’ zygosity and zygosity as derived from the questionnaire and DNA analyses at 8 and 29 months, were used to establish four groups for comparison: (1) parents who correctly classified their MZs as MZs (MZC); (2) parents who incorrectly classified their MZs as DZs (MZI); (3) parents who correctly classified their same-sex DZs as DZs (DZC); and (4) parents who incorrectly classified their same-sex DZs and MZs (DZI). This allowed for direct comparison of twin correlations between parents who misclassified and correctly classified MZ and DZ pairs. Scores for each of the BEBQ and CEBQ scales were regressed on age, sex and gestational age of the twins. Intraclass correlations (ICCs) were calculated and compared for each of the four separate groups and for the two time points (8 and 29 months) when data on the parents’ opinion regarding their twins’ zygosity was collected. Parental classification of zygosity at 8 months was used to compare the ICCs for the BEBQ scales; parental classification of zygosity at 29 months was used to compare the ICCs for the CEBQ-T scales. ICCs were calculated using SPSS Version 22 for Windows.

## Results

All opposite sex twin pairs were classified as DZ. Zygosity questionnaire data was collected for same-sex twin pairs at 8 months (SD = 2.1; n = 1586) and 29 months (SD = 3.3; n = 934). 934 families (58.9 % of all same-sex pairs) provided questionnaire results at both time points. For the majority of pairs (n = 827, 88.5 %) zygosity assignment matched across the two questionnaires. The Spearman correlation coefficient between the zygosity questionnaire classification at 8 and 29 months (n = 934) was 0.80 (p < 0.001) and the Kappa statistic (a measure of agreement) was also 0.80 (p < 0.001), indicating a good test–retest reliability. A total of 1127 families had provided DNA samples for both twins; of these, 81 pairs were randomly selected for zygosity testing.

107/934 pairs (11.5 %), who had questionnaire data at both time points, could not be conclusively allocated using the questionnaire data: 41 pairs had a mismatch of classification between the two questionnaire time points (MZ then DZ; or DZ then MZ); 59 pairs fell into the uncertain range at either 8 or 29 months (i.e. uncertain at 8 months, then MZ or DZ at 29 months; or, MZ or DZ at 8 months, then uncertain at 29 months); 7 pairs fell into the uncertain range at both time points. Therefore, where available, DNA was used to classify the zygosity of these pairs. DNA was available for 87/107 pairs, and the genotyping process was successful for 86/87 pairs (34/41 mismatches; 46/59 pairs who were uncertain at either 8 or 29 months; 6/7 pairs who were uncertain at both time points). There were also 24 pairs for whom questionnaire data was only available at 8 months, but for whom DNA was also available; for these 24 pairs DNA was used for zygosity classification.

Results from the questionnaire and the DNA testing were combined to provide the most accurate zygosity assignment for the Gemini sample. For 1239 pairs, questionnaire data only was used to allocate zygosity (n = 590 pairs with data at 8 months only; n = 636 pairs with data at both 8 and 29 months; n = 6 pairs with classification at 8 months but uncertain zygosity status at 29 months; n = 7 pairs with uncertain zygosity status at 8 months, but classified at 29 months). DNA was used to zygosity test (n = 310 pairs), including: a random sample of 81 pairs; 86 pairs for whom zygosity could not be classified conclusively using questionnaire data; 24 pairs who only had questionnaire data at 8 months; and 119 pairs whose parents requested a zygosity test.

A total of 749 twin pairs (31.2 %) were classified as MZ and 1616 (67.3 %) twin pairs were classified as DZ (including 816 opposite sex DZ twins), based on the questionnaire and DNA results. For a further 37 pairs (1.5 %) zygosity could not be established, as questionnaire results were unclear and no DNA was provided. A detailed list of the final zygosity classification in this sample can be found in Table 2.

**Table 2:** Detailed zygosity classification for the Gemini sample combining questionnaire data and DNA genotyping

	N	Classification procedure
Total	2402	
MZ	749 (31.2 %)	
	219	Using available DNA
	282	Matching questionnaire results
	248	Only questionnaire at 8 months available
DZ	1616 (67.3 %)	
	816	Opposite sex twin pairs
	91	Using available DNA
	354	Matching questionnaire results
	342	Only questionnaire at 8 months available
	6	Questionnaire at 8 months only, uncertain result at 29 months
	7	Questionnaire at 29 months only, uncertain result at 8 months.
Unknown	37 (1.5 %)	Uncertain questionnaire results, no DNA available

### Validation of the zygosity questionnaire using DNA

DNA from the random sample of 81 twin pairs was used to validate the zygosity questionnaire. DNA confirmed 43 pairs as MZ and 38 as DZ; which exactly matched the results of the questionnaires. Comparing the questionnaire results with *all pairs* for whom DNA was available showed high concordance between the two questionnaires with DNA. At 8 months, 279 pairs had both questionnaire classified zygosity and DNA; the 8 month questionnaire matched DNA results for 87.5 % of the sample. At 29 months, 248 pairs had both questionnaire classified zygosity and DNA; the 29 month questionnaire matched DNA results for 96.8 % of the sample.

### Misclassified zygosity

At 8 months there were 1528 pairs of twins who had *both* researcher-classified zygosity (using the questionnaires and DNA) *and* parent-classified zygosity (i.e. parents had responded to the question “do you think your twins are identical?”). There was high concordance between parental classification of zygosity and researcher measured zygosity (85.2 %). However 30.1 % (220/731) of parents of MZ twins mistakenly believed them to be DZ. Only six parents of same-sex DZ pairs mistakenly classified them as MZs (0.75 % of parents of same sex DZs, 6/797).

At 29 months there were 898 pairs of twins who had *both* researcher-classified zygosity (using the questionnaires and DNA) *and* parent-classified zygosity (i.e. parents had responded to the question “do you think your twins are identical?”). At 29 months 26.3 % of parents of MZs (119/453) misclassified them as DZs. Again the number of misclassified DZ twins was very low (2/445 same-sex DZ pairs). These analyses used only same-sex twin pairs; opposite-sex pairs (n = 816, 33.3 %) and pairs of unknown zygosity (n = 37, 1.5 %) were excluded. All percentages and numbers of twin pairs used in the analyses are shown in Table 3 for 8 and 29 months separately. Parental belief about zygosity was stable over time. Of the parents who responded at both 8 and 29 months, 94.9 % (852/898) held the same belief at both time points.

**Table 3:** Numbers and percentages of twin pairs for the different zygosity groups at 8 months and at 29 months

	8 months		29 months	
	Frequency (n)	Percent (%)	Frequency (n)	Percent (%)
Sample of same-sex twin pairs (excluding unknown zygosity)				
Total	1549		1257	
MZ	749	48.4	616	49.1
DZ	800	51.6	641	50.1
Zygosity groups according to parents’ beliefs of zygosity and zygosity derived from questionnaire and DNA data				
Total	1528^a^	100	898^b^	
MZC	511	33.4	334	37.2
MZI	220	14.4	119	13.2
DZC	791	51.8	443	49.3
DZI	6	0.4	2	0.2

*MZ* Monozygotic; *DZ* Dizygotic; *MZC* MZ pairs correctly classified as MZ by parents; *MZI* MZ pairs misclassified as DZs by parents; *DZC* DZ pairs correctly classified as DZ by parents; *DZI* DZ pairs misclassified as MZs by parents

^a^n is less than the total n for MZs (1549) because it only includes pairs with *both* classified zygosity at 8 months *and* pairs whose parents answered the question “do you think your twins are identical?”

^b^n is less than the total n for DZs (1257) because it only includes pairs with *both* classified zygosity at 29 months (using questionnaire and DNA data) *and* pairs whose parents answered the question “do you think your twins are identical?”

Furthermore 1427 parents stated that they were informed by a health professional about their twins’ zygosity, and the majority agreed with the health professional’s opinion (n = 1375; 96.4 %). Only a few parents (n = 52, 3.6 %) disagreed with the opinion of the health professional.

### Comparison of intraclass correlations

Intraclass correlations (ICCs) of eating behaviors measured by the BEBQ and CEBQ-T were calculated separately for the different zygosity groups, based on the parental belief at 8 months and 29 months, respectively.

#### Baby eating behavior questionnaire

Scores from the BEBQ were regressed on sex, gestational age and age of the children at questionnaire completion. Only six same-sex DZ pairs were misclassified as identical by the parents; because of the small sample size for these pairs the 95 % confidence intervals were wide and reliable ICCs could not be calculated. We therefore only report the results for three groups: MZC, MZI, and DZC.

Overall there was no difference in magnitude between the size of the ICCs for correctly and misclassified identical twins for any of the four eating behaviors. For SR, EF and SE the 95 % confidence intervals overlapped, indicating that the ICCs were not significantly different for MZC and MZI. The 95 % confidence intervals did not overlap for the ICCs for FR, however the difference in magnitude was very small (MZC, 0.89; MZI, 0.82) and the large sample size ensured that the 95 % confidence intervals were narrow, such that trivial differences were significant. Additionally, the ICCs for the DZC group were substantially smaller than those for the MZI group for all four eating behaviors, and none of the 95 % confidence intervals overlapped.

#### Child eating behavior questionnaire (Toddler)

A similar pattern of results was found for eating behaviors measured by the CEBQ-T at 16 months. For each of the five eating behaviors the magnitude of the ICCs for MZC and MZI was similar. For EF, SR, FR, FF and SE there was no significant difference between MZC and MZI, indicated by the overlapping 95 % confidence intervals. For EOE the 95 % confidence intervals did not overlap, but touched for the MZC and MZI groups. Again, the ICCs for the DZC group were substantially smaller than the MZI ICCs for each of the five eating behaviors, and none of the 95 % confidence intervals overlapped. All ICCs for the different zygosity groups and eating behaviors are presented in Table 4.

**Table 4:** Intraclass correlations for eating behaviors measured at 8 months (BEBQ) and 16 months (CEBQ-T) for correctly and misclassified zygosity

	MZC	MZI	DZC
*BEBQ 8 months*			
SR	0.84	0.80	0.51
95 % CI	0.81–0.86	0.75–0.84	0.45–0.56
n (pairs)	502	215	772
FR	0.89	0.82	0.60
95 % CI	0.87–0.91	0.77–0.86	0.55–0.64
n (pairs)	500	215	768
EF	0.80	0.80	0.47
95 % CI	0.76–0.83	0.75–0.85	0.41–0.52
n (pairs)	499	212	769
SE	0.82	0.82	0.40
95 % CI	0.79–0.85	0.77–0.86	0.39–0.46
n (pairs)	502	216	772
*CEBQ-T 16 months*			
SR	0.93	0.94	0.62
95 % CI	0.91–0.94	0.92–0.96	0.55–0.67
n (pairs)	308	113	413
FR	0.95	0.96	0.66
95 % CI	0.93–0.96	0.94–0.97	0.6–0.71
n (pairs)	308	112	412
EF	0.92	0.92	0.59
95 % CI	0.90–0.94	0.88–0.95	0.52–0.65
n (pairs)	308	113	413
FF	0.91	0.88	0.55
95 % CI	0.88–0.92	0.82–0.92	0.48–0.62
n (pairs)	308	113	413
EOE	0.98	0.99	0.90
95 % CI	0.97–0.98	0.98–0.99	0.88–0.92
n (pairs)	308	113	412
SE	0.88	0.88	0.43
95 % CI	0.85–0.90	0.84–0.92	0.35–0.50
n (pairs)	308	113	413

*MZ* Monozygotic; *DZ* Dizygotic; *SR* Satiety Responsiveness; *FR* Food Responsiveness; *EF* Enjoyment of Food; *FF* Food Fussiness; *EOE* Emotional Overeating; *SE* Slowness of Eating; *MZC* MZ pairs correctly classified as MZ by parents; *MZI* MZ pairs misclassified as DZs by parents; *DZC* DZ pairs correctly classified as DZ by parents; *CI* Confidence Interval

## Discussion

We used the ‘misclassification of zygosity’ design in a novel approach to test for parental bias in reporting of similarities in infant and child eating behavior among twin pairs. We showed for the first time that parents who misclassified their MZs as DZs nevertheless scored them as similarly as the parents who correctly classified their MZs as MZs, on a range of eating behaviors. Intraclass correlations were compared for misclassified and correctly classified MZ pairs for a range of eating behaviors, measured by widely used parent-report questionnaires for infants (the BEBQ) and toddlers (the CEBQ-T).

The results showed that the magnitude of the intraclass correlations was very similar across both correctly and misclassified identical twins. In addition, the intraclass correlations for the correctly classified DZs were markedly smaller than those of the incorrectly classified MZs, and none of the 95 % confidence intervals overlapped across the two groups. These results indicate that parents’ perceptions of their twins’ zygosity did not bias their scoring of their eating behaviors, insofar as they did not score their MZ twins less similarly if they mistakenly believed them to be DZ. The problem of parental rater bias is often raised in research with infants and children. These outcomes suggest that no parental bias was found in relation to zygosity status, and supports the validity of the twin method for establishing the genetic and environmental influences on eating behaviors in infants and toddlers.

### Implications

The twin method has been widely used to investigate the etiology of complex human behavior and constant critical analysis of the assumptions underlying this method contributes to its ongoing success. Previous studies used the misclassified zygosity methodology to test for violations of the equal environments assumption (EEA), confirming its overall validity (Felson 2014). This approach was also previously used to investigate the effect of self-reported zygosity on twin similarity of eating patterns in adulthood. Results showed that identical twins correlate higher than DZ twins on healthy eating patterns, regardless of their self-reported zygosity (Gunderson et al. 2006), indicating that measures of eating behavior can also be used reliably in adult twin samples. In comparison to previous misclassified zygosity studies (Goodman and Stevenson 1989; Kendler et al. 1993, 1994; Xian et al. 2000; Cronk et al. 2002; Gunderson et al. 2006; Conley et al. 2013), this research is, to our knowledge, the first attempt to utilize the design in a sample of infant and toddler twins to test for biases in relation to parental belief about zygosity.

As previously reported parents can be misinformed about the zygosity of their children (Ooki et al. 2004). In this sample, of 749 MZ twins, 220 (29.4 %) were misclassified as DZ by parents when the twins were 8 months old. Previous research suggests that parental misclassification of MZs as DZs often stems from false information given by health professionals (van van Jaarsveld et al. 2012). In this study the majority (n = 1375, 96.4 %) of parents agreed with the health professional’s opinion about their twins’ zygosity. These results might be seen as an indicator that parents trust health professionals and base their own opinion on the judgement of a professional. However many health professionals classify twin pairs as non-identical if a prenatal scan shows that they are dichorionic (each has their own placenta), regardless of the fact that approximately one third of MZ twin pairs develop with separate placentas (Hall 2003). Knowledge gaps of obstetricians and gynecologists in twin prenatal development is suggested to be the cause of the misinformation (Cleary-Goldman et al. 2005). Using reliable measures of zygosity determination in same-sex twins is crucial for twin research. Additionally, zygosity classifications are important for medical reasons, such as prenatal diagnosis of genetic disease or disorders and transplant compatibility, as well as the identity and social development of the children (Stewart 2000; Hall 2003).

### Limitations

In the current sample only a small number of same-sex DZ pairs were misclassified as MZ (n = 6 at 8 months; n = 2 at 29 months). Intraclass correlations were therefore often not significant and had wide 95 % confidence intervals, making them difficult to interpret and were therefore not included in the present analysis. A previous study of parental zygosity classification in 1244 Japanese families with twins born between 1960 and 2002 found a slightly higher (but still small) number of misclassified DZ twins (31/323 DZ pairs were misclassified as MZ). However, this study found higher rates of misclassification overall (Ooki et al. 2004). Future studies using the misclassified zygosity design would benefit from increased sample sizes to include more misclassified DZs. Larger samples would enable researchers to make comparisons between correctly classified and misclassified DZ twins, to provide more evidence in support of the validity of parental reports for the twin method.

For the majority of the sample zygosity was ascertained using a zygosity questionnaire sent to parents when the twins were 8 and 29 months old. When comparing questionnaire results collected at 8 months with all available DNA collected, zygosity ascertainment matched for 87.5 % of the sample. For data collected at 29 months the accuracy of the questionnaire was higher at 96.8 %; indicating that the questionnaire may be slightly more accurate for toddlers than infants. As children might become more distinct as they grow up, it seems reasonable that parent rated zygosity is slightly more accurate when the twins are older. Regarding these rates of accuracy overall, it is also important to acknowledge that DNA was only used to zygosity-test a subset of the sample that included twin pairs who were difficult to classify (pairs for whom there was a mismatch between the zygosity questionnaire results, and pairs whose parents requested a DNA test, implying that they were uncertain about their twins’ zygosity), as well as a random sample of 81 pairs. For the random sample only there was a 100 % match between the questionnaire and DNA zygosity classification. However, although we feel confident that zygosity can be accurately classified using a parental questionnaire for most twin pairs, DNA genotyping remains the gold standard for zygosity ascertainment and should ideally be available for more twin pairs. Nevertheless, zygosity testing using DNA remains costly and the use of questionnaire is more feasible for larger cohorts like Gemini.

This study only assessed parental bias in relation to eating behavior in infancy and toddlerhood. Additional studies using a similar design could investigate the parental bias on other parent rated child behaviors, such as physical activity and personality. It would also be useful to understand if parental bias starts to emerge as children mature and naturally become more different from another. Future studies using the misclassified zygosity design assessing parental bias in school-aged children would be useful.

## Conclusion

A potential flaw in the twin method is parental bias in reports of similarities in twin behavior, related to perceived zygosity. The outcomes of this study suggest that there was no parental bias related to zygosity in the Gemini twin cohort when parents reported on a range of infant and child eating behaviors.
